# Seasonal content of heavy metals in the “soil–feed–milk–manure” system in horse husbandry in Kazakhstan

**DOI:** 10.14202/vetworld.2021.2947-2956

**Published:** 2021-11-24

**Authors:** Nazym Kozhanova, Nurzhan Sarsembayeva, Bozena Lozowicka, Zhassulan Kozhanov

**Affiliations:** 1 Department of Veterinary Sanitary Examination and Hygiene, Faculty of Veterinary Science, Kazakh National Agrarian Research University, Almaty, Kazakhstan; 2 Institute of Plant Protection, National Research Institute, Bialystok, Poland; 3 Department of Technology of Production of Livestock Produce, Faculty of Technology and Bioresources, Veterinary Science, Kazakh National Agrarian University, Almaty, Kazakhstan

**Keywords:** feed, heavy metals, milk, soil

## Abstract

**Background and Aim:**

The quality of food, especially animal-based food, is crucial for human health. However, the quality of milk and other animal products has become an acute cause for concern in Kazakhstan. Technogenic dispersion of heavy metals (HMs) causes adverse effects on living organisms and creates unfavorable conditions for the existence of humans, animals, and plants. The purpose of this study was to analyze the content of several HMs in samples of soil, horse feed (hay, mixed feed, and bran), mare’s milk, and manure to assess bioaccumulation and possible adverse effects on the bodies of horses. An additional purpose was to identify areas with acceptable conditions for obtaining environmentally safe horse breeding products in the agricultural zones of the Almaty region, Kazakhstan.

**Materials and Methods:**

Samples were obtained from two farms in the Almaty region in 2020 (spring, summer, and autumn). In total, 72 soil samples were analyzed, which were taken from the upper humus horizon to the depth of the arable layer. Eighty-six samples were taken from the feed of horses. Green feed was represented by perennial and annual grasses (alsike clover, *Medicago sativa*, sweet yellow clover, as well as pea and oat mix). Barley and wheat bran stored in the warehouses of the farm were sampled for the research as feed supplements. The mixed feed comprised components such as maize and sunflower. In addition, 46 samples of mare’s milk and 28 samples of horse manure were collected. The HM analysis was performed in the laboratory of the Kazakh-Japanese Innovation Center. The residual amounts of HMs were determined using an absorption spectrometer with a voltammetric analyzer. The content of cadmium (Cd), lead (Pb), mercury (Hg), and arsenic (As) in all the studied samples of soil, feed, mare’s milk, and manure did not exceed the threshold limit values (TLVs), suggesting that the intake of these toxic elements into the human body with food was low.

**Results:**

The average Cd concentration was in the range of 0.29-0.31 mg/kg in soil samples and in the range of 0.20-0.27 mg/kg in feed samples. In milk, the Cd concentration varied from 0.01 to 0.02 mg/L and was lower in summer and higher in fall. The total average Cd content in horse manure was 0.1844 mg/kg. The concentration of Pb in soil samples ranged from 1.09 to 1.30 mg/kg with the lowest value in spring and the highest in fall. In the feed, the concentration of Pb varied from 0.14 to 0.76 mg/kg and in milk from 0.03 to 0.15 mg/L. The average concentrations of Hg and As in soil samples averaged 0.022 and 0.019 mg/kg, respectively, and were within the TLVs.

**Conclusion:**

In the study areas, the calculated transition rates in the soil–feed–milk–manure system revealed that the greatest transition of HMs was observed for Pb and Cd, and a smaller migration was observed for Hg and As. The tendency of accumulation of trace elements continued in the feed.

## Introduction

Horses are an integral part of Kazakh culture and history. The Ministry of Agriculture, Kazakhstan, maintains a program for the intensive development of horse breeding for the preservation and improvement of the gene pool of the Mugalzhar, Kushum, and “Kazakh Jabe” breeds and the breeding of pedigree horses of thoroughbred horse breeds. Local breeds are typically resistant to diseases such as piroplasmosis and necrobacillosis, which can cause great damage to horses of other breeds imported to Kazakhstan [1,2].

Paratypical factors greatly influence horse fertility, the most important of which is the level and quality of feeding. Clearly, feed and feed quantity should not endanger animal health [3,4]. Anthropogenic terrestrial pollution of numerous farms has led to changes in the natural composition of soils, reservoirs, and vegetation, which, in turn, negatively affects the reproductive functions of animals [5]. The most dangerous toxicants with a direct negative effect on humans and animals (e.g., through synergistic effects in the bodies) are heavy metals (HMs) [6]. HMs are a group of chemical elements with a density of more than 5 g/cm^3^ or a relative atomic mass of more than 40. Within this group, several metals are toxic. This group comprises mercury (Hg), arsenic (As), cadmium (Cd), and lead (Pb) [7]. HMs fundamentally change the intake of trace elements in plants that perform essential biochemical functions and that are organically associated with increasingan organism’s resistance to ionizing radiation. This behavior is relevant for the effects of radioactive contamination.

Hg residues originate from the substance’s use in fungicides [8]. Pb accumulating in the soil and plants originates mainly from the exhaust gases of internal combustion engines associated with traffic. Its toxic effect on plants begins to manifest at concentrations of approximately 5 mg/kg of soil. However, Pb compounds are harmful to plants at all concentrations [9]. Cd is characterized by high toxicity relative to soil organisms. Cd can act as zinc in numerous biochemical processes. It disrupts the functioning of enzymes associated with respiration and other physiological processes (carbonic anhydrase, various dehydrogenases, and phosphatases), as well as those involved in the protein (proteinase and peptidase) and nucleic metabolisms. This leads to high phytotoxicity of Cd. As a chemical analog of zinc, Cd replaces it, which is necessary for the phosphorization of glucose and the process of forming and metabolizing carbohydrates. Cd typically originates from the widespread use of phosphates in agriculture.

A widely accepted maximum concentration of Cd is in the range of 0.2-2.0 mg/kg of soil, depending on the present level of soil fertility [10]. Under comparable conditions, the level of Pb and Cd absorbance varies with plant species. For example, green onions, carrots, beets, cabbage, potatoes, and tomatoes typically exhibit increased accumulation of Pb and Cd [10].

However, in all cases, agricultural areas are exposed to local pollution originating from traffic. Thus, the contamination of soil cover occurs consistently through a semi-elementalcomposition of toxic substances. Agricultural produce grown on radionuclide-contaminated soils must therefore be analyzed regarding the contents of Pb, Cd, As, and Hg. HMs enter agricultural plants through the following substances: Sewage sludge, sewage, and household garbage. In general, in all countries, the main sources of anthropogenic atmospheric pollution are thermal and other power plants, the ferrous and nonferrous metallurgy industry, oil production, traffic, and the construction materials industry [11].

The purpose of this study was to determine the composition and quality of local key biological resources — soil, feed, and horse organism — in two agricultural sites in the Almaty region, Kazakhstan. Bioaccumulation of HMs and the impact on the organism of horses were assessed. In addition, areas with acceptable conditions for obtaining environmentally safe horse products were identified.

## Materials and Methods

### Ethical approval

The work was approved by the Bioethics Commission of the Kazakh National Agrarian Research University, and the analyses comply with the Code of Professional Ethics of Veterinarians, the ethical principles of animal research established by the European Convention for the protection of vertebrates used for experimental and other scientific purposes.

### Study period and location

The sampling of objects and the study of residual amounts of heavy metals in them were carried out from March to October 2020 at two different sites, each in a distinct region of the Almaty region.

Site 1: Alem Trade farm (KZ LLP) is located outside the industrial mountainous zoneapproximately 5 km from the city of Almaty.

Site 2: Aydarbayev farm is located in the Almaty region, Enbekshikazakhsky district, the village of Saimasai. The farm is located next to the central highway in an industrial area.

The climate in both regions is characterized by hot and rainy summers and humid winters. The average annual temperature and precipitation at the first and second sites are 19.5°C and 1,400 mm and 21.9°C and 1290 mm, respectively. Detailed data on horses (i.e., gender, age, breed, weight, and main use) and the farms were obtained at the sampling sites. Then, the animals were physically examined to determine the state of nutrition, degree of hydration, color of the mucous membrane, time of filling of the capillaries, as well as heart rate and respiration.

The horses from Site 1 were only females of the “Kazakh Jabe” breeds. The horses from Site 2 comprised both sexes of the “Mugaljar” breed (3-12 years, average 4.5 years). Each group comprised ten randomly selected clinically healthy animals, from which samples of feed and feed additives, milk, and manure were obtained 3 times per year (at the beginning of the spring, in summer, and in fall).

Grazing horses had free access to water and were fed hay and full-fledged horse food, which consists of feed rations that contain all the substances necessary for the animal and are also capable of ensuring its normal vital functions. The maintenance, feeding, and watering of the mares, as well microclimate parameters, were identical at both sites and corresponded to the zootechnical standards for this type of animal. The biochemical analyses of soil, feed, milk, blood, and manure were conducted in the laboratory of the Kazakh-Japanese Innovation Center, Kazakh National Agrarian Research University.

The content of HMs in the soil, plants, and feed was determined through conjugate analysis. The transition of HMs through the food chain was assessed through the refinement of their coefficients. Finally, the spectrum of toxicants (Pb, Cd, Hg, and As), their levels in relation to the TLVs (ratio), and the influence of soil conditions on the accumulation of HMs was evaluated. Through determining the quantitative and migratory nature of HMs in the food chain and identifying the influence of various factors on it, the accumulation and transition rates (TRs) of HMs in thesoil–feed–horse system of the mare were determined.

The TRs of HM from soil to food and the animal’s body were determined by the formula (1):


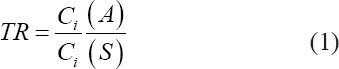


where, *TR* is the transition rate of the *i* – goelement from the daily ration to the milk of the animal; *C_i_*(*A*) – the content of the *i* – goelement in feed, manure (mg/kg), milk (mg/L); *C_i_*(*S*) – the content of the *i* – goelement in the soil, mg/kg.

Standard analyses of distinct variation series were performed using the generally accepted methods of variation statistics. The reliability of the differences was evaluated accordingly using the Student’s 1-criterion. The results of the analysis were considered statistically significant at p<0.05.

### Soil

To determine the levels of HMs in the soil, samples were taken from the upper humus horizon to the depth of the arable layer (0-30 cm) according to standard methods in soil science [12]. Soil sampling (400 g per sample) was performed 3 times per year: At the beginning of spring (after the snow had melted), in summer, and in fall (during harvest). The concentration of HMs was determined using the following method [13]: 2.0 g of the analyzed sample were placed in a glass beaker; 10 cm^3^ of nitric acid with a molar concentration of 0.5 mol/dm^3^ were added; the mixture was stirred and kept at a temperature of 90°C while continuing stirring for 3 h. The sample was then filtered through a paper filter into a 100 cm^3^ measuring flask. The volume was adjusted to 100 cm^3^ with bidistilled water. The resulting solution was analyzed for AAS.

The mass fraction of the element to be determined in the sample for each determination *(X_i_*^AAS^*)*, million^-1^, was calculated by the formula (2):


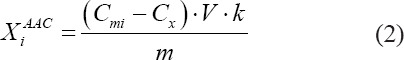


Where:

*C_Mi_* *–* Mass concentration of an element in the analyzed solution, found from the calibration characteristic, mg/dm^3^;

*C_X_* – Mass concentration of the element in the blank solution, found by the calibration characteristic, mg/dm^3^;

*V* *–* the volume of the analyzed solution, (*F* = 100 cm^3^);

*k –* dilution ratio (from 1 to 1000);

*t* – the weight of the sample weight, g.

### Feed

Feed comprised perennial and annual grasses (alsike clover, *Medicago sativa*, sweet yellow clover, as well as pea and oat mix). Samples were obtained from feed harvested directly on the farms that formed the basis for the feed ration. Samples of the mixed feed were obtained from the warehouse. Grasses from the pastures were collected monthly, from June to September, from 10 accounted patches of 1 m^2^, located diagonally on the plot. The grass was mowed at the height of 3-5 cm. All feed additives (wheat bran and barley), as well as mixed feed, which consisted of a mixture of corn and sunflower, were stored in the warehouses of the studied farms. Sampling from mixed feed and feed additives was carried out using the following method [14]: Spot samples were taken with a barn probe of objects. The entire surface of the feed was divided into squares of 4-5 m^2^ each. Samples were taken from the center of each square (at the height of the square of up to 0.75 m—from the upper and lower layers and at the height of over 0.75 m—from the upper, middle, and lower layers). From the mixed feed kept in closed bags point samples were taken with a bag probe from the top and bottom thirds of the bag. The probe was inserted with the groove facing downward, turned 180°, and then retrieved. Five percent of all bags were sampled. A total of 86 samples of animal feed were taken, of which: 25 samples from hay and freshly cut grasses, 9 samples from alfalfa, 8 and 23 samples from bran, and 21 samples of barley from the mixed feed. The raw materials were dried immediately after collection by air drying to avoid the destruction of substances. The conditions of collection and storage were identical for all feed samples.

The content of HMs was determined through the atomic absorption method according to the following method [15]: A sample weight of 10-20 g was placed in a crucible. Then, the crucible was placed in a cold muffle furnace, and the temperature was raised to 250-300°C and subsequently cooled. The ash was moistened with a few drops of distilled water; 5 cm^3^ hydrochloric acid solution was added, and then the mixture was diluted with distilled water at the ratio of 1:1. Next, the crucible was placed in a boiling water bath or electric stove and evaporated to achieve a wet state, preventing the spray and calcination of the sediment. A total of 10-15 cm^3^ of a nitric acid solution was poured into the crucible using a dispenser. The mix was diluted with distilled water at the ratio of 1:1; then, the crucible was covered with a watch glass and heated on an electric stove to boiling point and then kept in a boiling water bath for 30 min. After cooling, the ash solution was filtered into a volumetric flask with a capacity of 50 cm^3^ through a paper filter. The filter was first thoroughly washed with a solution of nitric acid diluted with distilled water at the ratio of 1:1. The crucible was rinsed several times with hot distilled water and drained onto the filter. The filter was thoroughly washed with distilled water. The volume of the solution in the flask was topped up to 50 c^m^3 with distilled water and mixed. The sediment was allowed to settle. The liquid above the sediment was carefully retrieved for analysis. Determination of the mass concentration of metals in the ash solution was performed using the following analytical lines (sensitive absorption lines of elements) with the following wavelengths, nm: Pb–217.0; Cd–228.8.

The mass fraction of the metal in the test sample (mg/kg) was calculated by the formula (3):


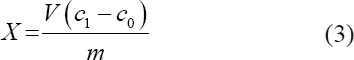


Where:

*c*_1_ – mass concentration of metal in the ash solution, found according to the calibration schedule, mg/dm;

*c*_2_ – mass concentration of metal in the solution of the control experiment, mg/dm;

*V* – the volume of the initial ash solution, cm;

*m* – sample weight, g.

### Milk

The volume of each milk sample ranged from 300 to 500 mL. Milk samples from the flasks were obtained using the following method [16]: A clean and dry tube was immersed in the milk and retrieved while closing the upper end with the thumb. The retrieved milk was poured into a clean and dry bottle, which was closed with a rubber stopper. Bottles with milk samples were labeled and stored in the refrigerator at a temperature of 4°C until the next day. A total of 36 milk samples were analyzed.

### Manure

Samples of horse manure were retrieved directly from the rectum through the anal opening using a customized bucket attached to a stick and stored in clean plastic bags. Horses were never touched, stimulated, or forced to excrete feces or urine. Equal amounts of horse feces were pooled, homogenized, and stored at −20°C. The concentration of Pb, Cd, Hg, and As in horse feces was determined by atomic absorption spectrometry.

### Measuring instruments and analysis

The quantitative determination of HMs (Cd, Pb, As, and Hg) was performed on a novAA350 atomic absorption spectrometer (Analytik Jena, novAA® 350, spectral range 185-900 nm, Jena, Germany) with a TaLab voltammetric analyzer (Tomsk, Russia).

### Sample preparation

Sample preparation was performed by dry and acid mineralization. Nitric acid solution (HNO_3_, Alita LLP, Kazakhstan) was added to the suspension of the feed, soil, and manure at the rate of 1 mL per 50 g of the product; the solution was then mixed, placed on an electric stove, and carefully charred until all smoke disappeared. Then, the bowl was placed in an electric furnace (muffle furnace PE-4820 [7.2l/1000°C, JSC Ekroschem, Saint Petersburg, Russia]) preheated to approximately 250°C. Simultaneously, the bowl with the sample suspension was heated using an infrared lamp. Then, ethyl alcohol was added to the bowl containing the sample at a rate of 5 mL per 1 g of dry matter; it was then covered with a watch glass and incubated for 36 h. Next, charring was performed. After charring, the mineralization of samples was performed in an electric furnace by increasing the temperature gradually (by 50°C every 30 min) to 450°C. Mineralization continued at this temperature until gray ash was obtained. The bowl with ash was retrieved from the electric furnace after 10-15 h of ashing, cooled down to 15-18°C, and then, the content was moistened by drops with 2-3 drops of nitric acid solution. The acid was entirely evaporated in a water bath; the next incubation in a desiccator was performed at 140°C. After cooling, the bowl with the sample was placed in the cooled electric furnace again. The temperature was gradually increased to 300°C, and the sample was incubated for 0.5 h. This cycle was repeated three times in duplicate [17].

To determine the concentration of Pb and Cd, the ash was dissolved in a crucible by heating it in nitric acid (1:1) by volume at a rate of 1-5 cm^3^ of acid per sample, depending on the ash content of the product. The solution was evaporated to wet salts. The precipitate was dissolved in 15-20 cm^3^ of nitric acid (mass fraction of 1%), transferred quantitatively to a 25 cm^3^ volumetric flask, and topped up to 25 cm^3^ with the same acid. Then, the spectrometer was prepared for operation and the measurement conditions were selected [18].

The mass concentration of As in the sample was determined by adding a certified mixture of As (III) to the analyzed solution. The total concentration of As was determined after reduction of As (V) compounds to As (III) with sodium pyrosulfite or sulfuric acid hydrazine during evaporation of the sample in the presence of sulfuric acid. To determine the content of As (V) in the milk, the sample was evaporated to dryness with the addition of concentrated hydrochloric acid (HCl, Alita LLP, Kazakhstan). The dry residue was incubated in a furnace (muffle furnace PE-4820 [7.2L/1000°C, LLC Ekroschem]) at >450°C. As (V) preserved in the sample was reduced to As (III) by sodium pyrosulfite; then, As (III) was determined in the mineralization product. The mass concentration of As (III) was defined as the difference between the total As concentration and the mass concentration of As (V) [19].

The mass concentration of Hg was determined by inversion voltammetry after pretreatment of the sample through ozonation (1.5-2.5 min). The ozonation of samples was performed directly in the cells of the analyzer with the use of an ozonizing device Chisto-TA (attachment to voltammetric analyzer TA-Lab, LLP Elementum, Kazakhstan). The mass concentration of Hg in the test samples was determined by adding certified mixtures with a specified content of Hg [20]. Analyses were performed in two repetitions.

The concentrations of the detected elements in the samples were calculated using the metrological processing of the results of the subsequent method [17]: Under the conditions of repeatability, two groups of measurements were performed – the first group, which gave *n_1_* measurement results with an arithmetic mean of ȳ_1_, and the second group, which gave *n_2_* measurement results with an arithmetic mean of ȳ_2_, and the standard deviation of the difference (ȳ_1_- ȳ_2_) was the following (4):


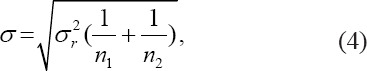


the critical difference for (ȳ_1_- ȳ_2_) was calculated as follows (5):


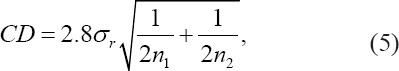


at a 95% probability level.

Quantitative indicators of the research results were processed by variational and statistical analysis using the Microsoft Excel, Statgraf, and Statgraf Plus software packages.

## Results

### Soil

The entry of HMs into the soil occurs through atmospheric sediments, metal-containing pesticides (Hg), and fertilizers [18]. Table-1 presents the gross contents of HMs at the study sites.

**Table-1:** Average content of heavy metals in soil samples by season, mg/kg (M±m).

Season	Economy	Heavy metals, TLV (mg/kg)			

		Cadmium	Lead	Arsenic	Mercury
			
		1.0	3.2	2.0	2.1
Spring	No. 1	0.0501±0.1	0.1549±0.1	0.0450±0.4	0.0311±0.2
	No. 2	0.1641±0.5	0.2434±0.11	0.0429±0.6	0.0661±0.31
Summer	No. 1	0.0869±0.4	0.0395±0.32	0.0062±0.1	0.0094±0.12
	No. 2	0.1039±0.14	0.0396±0.15	0.0078±0.3	0.0115±0.02
Fall	No. 1	0.7237±0.1	3.0636±0.02	0.0059±0.12	0.0083±0.2
	No. 2	0.6794±0.02	3.6301±0.4	0.0088±0.14	0.0112±0.1
Average number	No. 1	0.2869±0.31	1.086±0.14	0.0190±0.15	0.0163±0.4
	No. 2	0.3158±0.21	1.3043±0.13	0.0198±0.3	0.0296±0.6

The average content of Cd in the soil samples from Site 1 was 0.0501±0.1 mg/kg, 0.114±0.4 less than that observed in the samples from Site 2. In summer, the value for samples from Site 1 was 0.017±0.1 mg/kg. By contrast, in autumn, the value was higher, 0.0443±0.08 mg/kg. The average concentration of Cd in the soil of Site 1 was 0.2869 mg/kg, which does not exceed the respective TLV for absolutely dry soil matter. The soil from Site 2 contained on average Cd at 0.3158 mg/kg. The average amounts of Pb were 1.086 and 1.3043 mg/kg in the soil of Sites 1 and 2, respectively.

The annual average Hg concentrations in the soils of Sites 1 and 2 were 0.0163 and 0.0296 mg/kg, respectively, both of which do not exceed the TLVs. At Site 2, the largest share of the tested toxic elements was exhibited by Cd with 16.4%. The amount of Hg in the soil of Site 2 was 20.9% higher as compared to Site 1. These findings reveal that the research sites exhibited different degrees and combinations of contaminants.

The sample analysis revealed that the levels of HMs were close to the existing background values. In addition, an excess of Pb content was observed in samples obtained in fall. The samples of the research sites were characterized according to the ratio of HMs concentration to the corresponding TLVs; accordingly, the increase progressed as follows: Pb>Cd>Hg>As (1.195, 0.301, 0.022, and 0.019, respectively). Our results indicate that the contents of Hg, Pb, Cd, and As in the soils of the study sites did not exceed the TLVs.

### Feed

In plants, HMs are unevenly distributed in organs and tissues. The level of HM accumulation in the reproductive organs of plants is markedly lower than that in the vegetative ones. Accumulation is generally determined by the biological characteristics of the plant, the physiological role of the element in question, its content in the soil, and the abundance of plants. Knowledge regarding the specific pattern of accumulation of HMs in feed can aid in limiting their uptake into the body of fed animals and subsequently humans and so aid in minimizing adverse consequences [19].

Thus, as presented in Table-2, the Cd and Pb content in the studied areas was lower than the background values, but the Pb concentration at Site 1 in fall was 2.5-3 times higher than those in spring and summer. Regarding the feed of farm animals, standards for the content of HMs has been established. These were adopted as the TLVs for various types of feed (coarse and juicy, grain feed) in this study [20]. The observed contents of HMs in various types of feed (hay, fresh-cut grass, and mixed feed) in the study areas indicate a change in the indicators between the two surveyed farms within narrow limits: From 0.2055 to 0.2767 mg/kg for Cd and from 0.1407 to 0.7604 mg/kg for Pb (Table-2).

**Table-2:** The concentration of HM in horse feed produced in two farms of the Almaty region.

Farm No.	Sampling season	Type of feed	HM concentration in the feed, mg/kg			

			Cadmium M±m	Lead M±m	Arsenic M±m	Mercury M±m
1	Spring	Hay	0.3691±1.2	0.1463±0.13	±	-
		Mixed feed	0.2456±1.3*	0.1523±0.5	-	-
		Wheat bran	0.1562±0.1	0.1142±0.6*	-	-
	Summer	Grass clipping	0.2744±0.7	0.1771±0.03	-	-
		Barley	0.1621±1.3*	0.1064±0.8*	-	-
		Mixed feed	0.2521±0.8	0.1431±1.2*	-	-
		Alfalfa	0.2042±0.1	0.1548±0.4	-	-
	Fall	Bran	0.3851±0.6	0.1348±0.3	±	-
		Hay	0.4198±0.2	0.1376±0.1	-	-
	Average quantity		0.2767±0.03	0.1407±0.1	±	-
2	Spring	Hay	0.1521±0.2	0.0862±0.1	-	±
		Mixed feed	0.1548±0.1	0.1062±0.2	-	-
	Summer	Alfalfa	0.1137±003*	0.0476±0.8*	-	-
		Barley	0.1632±0.1	0.0588±0.01	-	-
		Hay	0.0426±1.2*	0.0722±0.2	-	-
		Mixed feed	0.1428±1.4*	0.0922±0.4	-	-
	Fall	Hay	0.5811±0.1	2.86±0.24*	-	±
		Mixed feed	0.2935±0.6*	2.76±0.3	-	-
	Average number		0.2055±0.8	0.7604±0.02	±	±
TLV			0.3	3.2	0.5	0.05


* p<0.05; (±) – trace of an element. TLV=Threshold level value, HM=Heavy metals

The concentrations of As and Hg in all types of feed were insignificant. In other words, only traces of these elements were found. In addition, the maximum observed Pb content was typical for feed produced at Site 1. At both sites, the Pb content in pasture grasses increased toward the end of the vegetation period. Still, the observed values of Pb and Cd were below the corresponding TLVs. In general, the feed produced at Site 2, which is located in a nonindustrial area of the Almaty region, exhibited the lowest contamination with HMs.

In animal husbandry, feed additives are typically used to improve weight gain, feeding efficiency, and the pigmentation of animals [4]. According to Zhou *et al*. [21], Cd is not added as a feed additive for animal growth. Cd is often present in mineral additives such as phosphates, Zn sulfate, and Zn oxide as an impurity. Hence, Cd can enter into the animal body through such feed ingredients. According to Li *et al*. [22], the average Cd content in feed for cattle was 2.79 mg/kg. We obtained data that a high amount of cadmium is not only found in animal feed but can also accumulate in sufficient quantities in animal byproducts [23].

In the feed samples from Site 1, the average amounts of Cd were 0.2569±0.86 mg/kg in spring, 0.2232±0.12 mg/kg in summer, and 0.4025±0.4 mg/kg in autumn. Average values of Cd in the feed samples at Site 2 averaged 0.1534±0.15, 0.1156±1.13, and 0.4373±0.35 mg/kg in spring, summer, and autumn, respectively. The highest Cd content was observed in the feed produced at Site 1.

At the same time, the ratios of HM concentrations to the corresponding values of MPC for different feeds of farm №1 change as follows:

- Hay (Cd: Pb: As: Hg=0.39: 0.14:±:-);
- Mixed feed (Cd: Pb: As: Hg=0.25: 0.15:-:-);
- Bran (Cd: Pb: As: Hg=0.27: 0.12:±:-);
- Fresh grass (Cd: Pb: As: Hg=0.27: 0.18:-:-);
- Barley (Cd: Pb: As: Hg=0.16: 0.11:-:-);
- Medicago (Cd: Pb: As: Hg=0.20: 0.15:-:-).

An excess of Cd concentration over the corresponding MPC values in the feed and feed supplements at Site 1 was observed. The Pb content in the feed (hay, mixed feed) at Site 1 in terms of excess ranked second. Its amount was within 0.11-0.18 mg/kg, respectively. Thus, controlling the content of Pb and Cd in the pasture grasses at this site is necessary. In addition, it is necessary to identify other types of HM concentrators (and primarily Pb).

### Milk

HMs were detected in the samples of mare’s milk. Cd and Pb were measured in various concentrations, whereas Hg and As were not detected. The highest amount of Pb (0.3831 mg/L) at Site 2 was observed in fall (Table-3). The average amount of Cd and Pb in the milk samples for the entire study period was 0.0053 mg/L. The increases in the concentration of Cd and Pb in winter and summer were accompanied by an increase in their content in the feed and also depended on the soil and climatic conditions.

**Table-3:** Content levels of HM in samples of mare’s milk (saumal) of the studied farms, M±m.

Number of HM, mg/L	Threshold level value	Economy					

		No. 1			No. 2		
	
		Spring	Summer	Fall	Spring	Summer	Fall
Cadmium	0.03	0.0153±0.1	0.0048±0.02	0.0058±0.2	0.0132±0.02	0.0070±0.13	0.0201±0.4
Lead	0.1	0.0816±0.03	0.0124±0.1	0.0053±0.4	0.0652±0.5	0.0149±0.1	0.3831±0.02
Lead	0.1	-	-	-	-	-	-
Mercury	0.05	-	-	-	-	-	-

–=Not found, HM=Heavy metals

Boudebbouz *et al*. [24] reviewed 60 original articles published since 2010 reporting Pb levels in raw cow’s milk worldwide. The highest levels of Pb (60 mg/L) were reported in an area consisting of granite and granite gneiss in India. Shahbazi *et al*. [25] reported that the accumulation of heavy and toxic metals in milk and dairy products may depend on the location of the farms. Numerous studies have assessed the content of metals in milk from different regions.

The concentration of Pb in milk differs according to country and local contexts. For example, the TLVs of Pb in cow’s milk are 0.05, 0.02, and 0.1 mL/L, in Germany and Holland, Turkey, and Russia, respectively [26]. The concentration of As in milk varies depending on the sampling location [27].

The content of Pb and Cd in mare’s milk at Site 1 did not exceed the MPC, and the average indices were: Cd 0.0153±0.01 mg/L, Pb 0.0816±0.03 mg/L; Cd 0.0048±0.02 mg/L, Pb 0.0124±0.1 mg/L; and Cd 0.0058±0.2 mg/L, Pb 0.0053±0.4 mg/L in spring, summer, and autumn, respectively. The content of HMs in milk at Site 2 did not exceed the MPC; the average of each element was Cd 0.0132±0.02 mg/L, Pb 0.0652±0.5 mg/L; Cd 0.0070±0.13 mg/L, Pb 0.0149±0.1 mg/L; and Cd 0.0201±0.4 mg/L, Pb 0.3831±0.02 mg/L in spring, summer, and autumn, respectively. Of all the samples tested for environmental safety, the maximum amount of HMs (Cd, Pb) was detected in the milk of Site 2. The concentration of elements increased in the following order: Pb>Cd>As=Hg (0.154:0.013:Nil: Nil).

### Manure

Numerous studies have examined the content of various metals in pig and cattle manure [28,29]. By contrast, few studies have examined the feces and urine from horses, which are economically less important. Table-4 presents the content of four HMs (Cd, Pb, As, and Hg) in the manure of various farms. In this study, the Cd and Pb content in horse manure was 0.1745-0.2044 and 0.1246-0.6445 mg/kg, respectively. The total average content of Cd and Pb in the surveyed farms of the Almaty region was 0.1844 and 0.3845 mg/kg. As in the feed, the highest metal content in cattle manure comprised Cd and Pb. In a previous study [28], significant correlations between Cd concentrations in animal feed and manure were reported. Thus, the addition of Cd to animal feed Pb to high Cd residues in the manure, which creates a high risk of contamination of agricultural land [30]. Besides Pb, no significant differences were found in the contents of HMs at Site 2.

**Table-4:** The average content of heavy metals in horse feces samples, mg/kg.

Heavy metals	Farm No. 1 n=10	Farm No. 2 n=10
Cadmium	0.1745±0.03	0.2044±0.05
Lead	0.1246±0.1	0.6445±0.04
Arsenic	±	±
Mercury	-	±

–=Not found

The content of HMs in animal manure largely reflects the content of HMs in the feed consumed by the animals and the efficiency of animal feed conversion. The use of As-containing additives in animal feed has led to the appearance of various As residues in manure [31]. In Austria, the average As content in cattle manure was 0.44 mg/kg [32], which is substantially higher than the content observed in this study. In our study, only traces of As and Hg were observed in horse manure.

### TRs of HMs in the soil–feed–milk–manure system

Table-5 presents the data on the migration of HMs in the soil–feed–milk–manure system. On the basis of the content of HMs in the soil and their accumulation in the feed, milk, and manure of mares, the TRs of HMs were calculated. These allow predicting the intake of toxic elements and aid in developing measures aimed at reducing the intake of toxic elements by adjusting the diet or using sorbents. TRs varied widely and depended on the characteristics of the metabolism of toxic elements in the body of the animals. The TR value in the soil–feed–milk–manure system was highest for Cd and lowest for Pb.

**Table-5:** Comparative assessment of the transition rates of heavy metals in the system “soil–feed–mare’s milk–manure.”

Heavy metals	Metal content level						

	In the soil of pastures	In feed, mg/kg		In milk, mL/L		In manure, mg/kg	
			
	Average content, mg/kg TR, %	Average content, mg/kg	TR, %	Average content, mg/l	TR, %	Average content, mg/kg	TR, %
Farm No. 1							
*Cd*	0.2869	0.2767	0.96	0.0086	0.03	0.1745	0.6
*Pb*	1.086	0.1407	0.12	0.0331	0.03	0.1246	0.11
*As*	0.0190	0*	-	0	-	0*	-
*Hg*	0.0163	0	-	0	-	0	-
Farm No. 2							
*Cd*	0.3158	0.2055	0.65	0.0134	0.06	0.2044	0.64
*Pb*	1.3043	0.7604	0.58	0.1544	0.2	0.6445	0.49
*As*	0.0198	0*	-	0	-	0*	-
*Hg*	0.0296	0*	-	0	-	0*	-

* 0=Minor traces of the element, (–)=No transition rate. Cd=Cadmium, Pb=Lead, As=Arsenic, Hg=Mercury, TR=Transition rate

A comparative assessment of the transition of HMs from the diet to the body of horses revealed that the migratory amounts of Cd ions were significantly reduced (in the case of the transition to manure, by 28.4%). Pb ions actively migrate to animal manure, which was clearly illustrated by a slight decrease in the coefficient by 8.3%. The migration of elements such as Hg and As from the diet to the product was less affected by the technology of harvesting feed (mowing, selection of feed, pressing, transportation, storage, compaction, sealing, and introduction of biological products or feed additives). The migration of elements from diet to milk was influenced not only by different preparation but also by the agroclimatic conditions of feed cultivation. The migration of Pb to milk was the lowest for the diet provided at Site 1 (by 0.03%). Regarding the rations of Site 1, the TRs of HMs, such as Cd and Pb, in milk were high (on average by 0.13%). By contrast, no TRs were observed for Hg and As.

The calculated transition coefficients in the soil–feed–milk–manure system indicate that the greatest transition of HMs was exhibited by Cd; slightly less migration was observed for Pb. At Site 1, the average Cd TR was 0.53% and that for Pb was 0.09%. At Site 2, the average TR for Cd was 0.45% and that for Pb was 0.42%. Transition coefficients for As and Hg were not calculated, because only traces of these elements were observed in the samples. In the feed and feed supplements, the tendency of accumulation of trace elements persisted. The analysis of the content of HMs in the samples revealed that the distribution occurred in descending order: Cd>Pb>As=Hg.

## Discussion

The statistical analysis revealed a considerable difference in the concentration of Cd and Pb in the samples of soil, feed, milk, and horse manure and depending on the sampling time. Seasonal variations in Cd content were significant. In the soil samples, the average Cd concentration was in the range of 0.29-0.31 mg/kg for all seasons. Although the Cd levels in the soil were lower in spring and higher during fall, the observed levels were lower than those reported by Zhou *et al*. [21]. The observed Cd concentration corresponded to the TLVs. Potential sources of Cd in the soil are the use of synthetic fertilizers and the presence of household waste in the water.

The concentration of Pb in the soil samples was 1.09-1.30 mg/kg with the lowest value in spring and the highest in fall. The recorded level of Pb in the soil samples was considerably lower than the concentration reported by Martin *et al*. [33]. The average annual concentrations of Hg and As in the soil samples at the two sites averaged 0.02239 and 0.0194 mg/kg, respectively, and did not exceed the TLVs.

In the feed samples, the amount of Cd was in the range of 0.20-0.27 mg/kg, exhibiting a lower value in spring and a higher value in fall. According to Adamse *et al*. [34], feed is mainly of plant origin and has a low level of HMs. However, when plants grow in a highly polluted environment; the concentration of HMs in plant-derived feed materials may increase either because of plant uptake or due to soil particles adhering to the plants, which can cause increased levels in the feed [34]. However, the observed Cd concentration in the hay samples was higher than the TLVs in fall (0.58 mg/kg). This level can cause the poisoning of livestock. Higher Cd concentrations in feed samples can be caused by ingestion of feed grown in Cd-contaminated soil as well as by grazing near roadways and contaminated water.

The concentration of Pb in the feed varied from 0.14 to 0.76 mg/kg throughout the study period, with the lower concentrations in spring and summer and higher concentrations in fall. According to Dai, high concentrations of Pb were observed in mineral feed additives that are used to enrich the main animal feed [35]. However, the Pb level in the feed samples at Site 1 was closer to the TLV in fall (2.8 mg/kg). The concentrations of As and Hg in all the samples of feed were insignificant. In other words, only traces of these elements were observed.

In the milk, the Cd concentration varied from 0.01 to 0.02 mg/L and was lower in summer and higher in fall. These values were significantly lower than those reported by Shahbazi *et al*. [25]. The observed Cd values in milk were almost the same as those reported by Eleboudi *et al*. [36]. The TLV for Cd content in milk samples is 0.03 mg/L, and the results obtained in this study were lower for all seasons.

The Pb concentration in the milk samples varied from 0.03 to 0.15 mg/L. The lowest concentration of Pb was recorded in the summer and the highest in fall. Literature on HM in the food chain is scarce. According to Christophoridis *et al*. [37], the content of HMs in milk and dairy products varies significantly depending on the season of the year. In general, the Pb level in the milk samples did not exceed the TLV of 0.1 mg/L.

HMs can be deposited in agricultural soil through manure. Adding organic waste to agricultural soils increases organic matter, contributes nutrients, improves the soil structure, and increases nutrient uptake by plants, which improves soil fertility and quality [38]. Despite the significant fertilizing value of the suspension, it can be rich in Zn, Cu, and other HMs obtained as a result of animal consumption [29]. The total average Cd content in horse manure at the study sites was 0.1844 mg/kg. As in the feed, the highest abundance of HMs was exhibited by Cd.

The Pb concentration in the manure samples ranged from 0.12 to 0.64 mg/kg. The lowest Pb value in the feed was observed in spring and the highest in fall. The use of phosphorous fertilizers for crop production may affect the Pb content in the feed. Consequently, the Pb level may be higher in the manure. Proximity to traffic can also cause an increase in the Pb levels in soil and feed, which also increases the level of Pb in manure.

According to the literature, the Almaty region currently exhibits a low level of environmental pollution [39]. The reasons for this are both the geographical location of the region and a low level of the environmental impact of local industry, transport, and agriculture [39]. Our findings suggest that in the studied administrative districts of the Almaty region, the calculated TRs in the soil–feed–milk–manure system indicate that the greatest transitions of HMs were for Pb and Cd, and smaller migrations were observed for Hg and As.

Therefore, the data indicate that the production of environmentally safe dairy raw materials is possible only if systematic control of the farm environment is ensured. Clearly, farms should not be contaminated with toxic elements. Nevertheless, studies like this provide useful data for guiding future initiatives for monitoring and assessing environmental pollution.

## Conclusion

The results of this study allow expanding the scientific foundations of horse breeding, including increasing the fertility of local horse breeds. The established correlations and coefficients allow predicting the amount of TM in feed, and this enables regulating their availability to animals. The comparative analysis in this study of the actual content of toxic elements in the feed and their TLVs suggests that the farms of the Almaty region have the opportunity to produce environmentally safe animal products. The findings of this study can be used in the development of practical regulatory documents on the use and rehabilitation of agricultural land with increased contamination with HMs to create an appropriate feed base.

## Authors’ Contributions

NK: Conception and design of the study, statistical analysis, and drafted the manuscript. NS: Conception and design of the study. BL: Revised the manuscript critically. ZK: Acquisition of data. All authors read and approved the final manuscript.
